# Nanoparticle-assisted gene editing for genomic disorders in the central nervous system

**DOI:** 10.4103/NRR.NRR-D-25-00766

**Published:** 2025-09-29

**Authors:** Hyeon-Yeol Cho, Jeong-Woo Choi

**Affiliations:** Department of Bio & Fermentation Convergence Technology, Kookmin University, Seoul, South Korea; Department of Chemical and Biomolecular Engineering, Sogang University, Seoul, South Korea

Genomic disorders affecting the central nervous system (CNS) are among the most complex and devastating conditions in human health. Moreover, these disorders, such as Rett syndrome, spinal muscular atrophy, and Fragile X syndrome, are typically caused by mutations in genes essential for neural development, synaptic function, or cellular homeostasis. Despite the genetic diversity involved, these diseases share key pathological features, including progressive neurodegeneration, disruption of neural circuits, and loss of cognitive or motor function. Meanwhile, one of the significant clinical challenges in treating CNS disorders is the limited regenerative capacity of the adult nervous system, which makes reversing disease progression extremely difficult once symptoms appear. In addition, the blood–brain barrier (BBB) restricts the passage of most systemically administered therapeutics, further complicating effective intervention. Consequently, current treatment options remain largely palliative, and effective cures remain elusive.

Until recently, therapeutic efforts for CNS disorders have focused on small-molecule drugs that modulate downstream signaling pathways or neurotransmitter levels. Nonetheless, while these interventions may alleviate symptoms in some patients, the underlying genetic causes remain unaddressed. However, advances in gene therapy have introduced new therapeutic possibilities. Indeed, viral vector systems, particularly adeno-associated viruses, have emerged as a favored method for delivering corrective genes or silencing mutated transcripts thanks to their broad tropism, favorable safety profile, and capacity for sustained expression (Wang et al., 2024). Nevertheless, the clinical application of these vector systems is hindered by limited cargo capacity and the potential for immune responses and inflammation. For example, eladocagene exuparvovec (Upstaza) received approval from the United States Food and Drug Administration in 2024 for the treatment of AADC deficiency. This therapy directly infuses adeno-associated viruses into the putamen to stimulate dopamine production and improve motor function. Follow-up histological studies have shown that comparable adeno-associated viruses doses activate p53-dependent DNA damage signaling and glial inflammatory responses in both rodent brains and primary human neurons (Costa-Verdera et al., 2025), raising concerns about long-term safety.

The design of clustered regularly interspaced short palindromic repeats (CRISPR) cargo for non-viral delivery systems has been extensively investigated, with numerous studies highlighting the potential of nanoparticle-based platforms to overcome the limitations inherent to viral vectors. Nanoparticle-based delivery technologies have advantages over virus-based technologies, as they do not impose strict cargo size restrictions, avoid permanent genome integration, and can be rapidly reconfigured to accommodate guide RNA modifications (Karmaker et al., 2025). When applying these strategies to genome editing, it is crucial to first define the therapeutic objective—whether it involves the precise replacement of the mutated gene region, correction of a point mutation, or the elimination of a deleterious gene. Such strategic considerations directly influence the composition of the CRISPR cargo payload. Fundamentally, all approaches require the delivery of either Cas9 or a base editor, with a single guide RNA, to facilitate nuclear localization for gene knockout or base editing. In the case of homology-directed repair, an additional component, a donor DNA template, is required. The effectiveness and suitability of each component can vary depending on the delivery modality. For example, rapid and transient nuclease activity can be achieved by delivering pre-assembled ribonucleoprotein complexes or messenger RNA. In contrast, longer-lasting expression can be attained via the delivery of DNA constructs, including the ~6-kb prime-editing machinery that exceeds typical adeno-associated virus packaging limits..

To accommodate these diverse CRISPR CNS components, nanoparticles needed to be functionalized to load nucleic acids and protect the cargo. Inorganic nanoparticles, such as gold nanoparticles and mesoporous silica nanoparticles, have been widely utilized for CNS gene therapy due to their ability to be easily functionalized with various ligands for delivery (Yang et al., 2023). However, concerns have been raised about the long-term accumulation of inorganic nanoparticles in the brain and the potential damage resulting from their inability to be readily cleared. To address these concerns, biodegradable nanoparticles, including lipid nanoparticles (LNPs), dendrimers, DNA origami carriers, and exosomes, were utilized (Lin-Shiao et al., 2022; Palanki et al., 2023). Especially, LNPs have gained prominence due to their successful application in mRNA vaccines and their demonstrated potential for CNS gene therapy.

Furthermore, both high encapsulation efficiency and timely cytoplasmic release are essential for the delivered cargo to work properly. Ionizable LNPs typically encapsulate 90% of a single guide RNA and 75% of a messenger RNA (Leung et al., 2015). Meanwhile, dendrimers and DNA origami carriers achieve similar loading with ribonucleoproteins by utilising multivalent electrostatic binding (Xie et al., 2025). Endosomal escape motifs such as protonated sponge polymers, pH-cleavable linkers, or membrane-active peptides, trigger release within six hours after cellular uptake. Additionally, exosomes can be engineered to transport nucleic acids (100 bp–20 kb) or proteins with minimal immunogenicity (Lu et al., 2023). Compared with LNPs, exosomes trigger less complement activation and reduced pro-inflammatory cytokine release, but their large-scale production and batch variability remain unresolved issues. Importantly, biodegradable platforms have also allowed repeated dosing in rodent models with minimal complement activation, directly addressing the neutralizing-antibody barrier seen with viral vectors (Lee et al., 2023).

Based on the discussion of CRISPR cargo design, the next challenge is to direct nanovehicles across the BBB. Particles smaller than 5 nm can diffuse through tight junctions in endothelial cell membranes, but their payload is extremely limited. Alternatively, receptor-mediated transcytosis can be utilized to transport particles as large as 20–120 nm across the BBB into the brain (Ding et al., 2020). Particles decorated with substances that have receptors on the BBB, such as glucose, transferrin, apolipoprotein E mimetics, or insulin ligands, can more efficiently “hitchhike” through the endogenous transport pathways of these substances and enter the brain parenchyma (Liu et al., 2025). A study has also been reported on temporarily loosening the tight junction proteins of the BBB by inserting aromatic small molecules such as borneol and menthol into the lipid membrane to increase permeability (Liu et al., 2024). Additionally, physical methods can be used to deliver nanoparticles loaded with CRISPR cargo into the brain. A single low-pressure focused ultrasound pulse has been shown to temporarily loosen the BBB, increasing local deposition in the affected area by approximately 5.5-fold compared to passive delivery (Kwak et al., 2024). An exceptional method was attempted to pass the BBB by the in utero intracerebroventricular injection of LNPs loaded with mRNA in fetal mice. This method resulted in over 40% of cortical neurons and 60% of hippocampal neurons being edited 10 weeks after birth (Gao et al., 2024). Another study demonstrated that adenosine diphosphate–LNPs carrying Cas9 mRNA and gRNA induced indels in 21% of brain cells when administered to neonatal mice within 7 days of birth; therefore, these data demonstrate the precision and potential of this method. Consequently, recent studies have proposed methods to safely and repeatedly pass the BBB and deliver CRISPR cargo to the brain by modifying the surface of nanoparticles using various principles. However, it is still challenging to provide sufficient editing material to correct all cells that require gene correction.

Despite progress in BBB penetration, directly editing genes within the brain remains associated with off-target risks and potential long-term toxicity. An alternative approach that is gaining attention involves gene-edited stem cells prepared using nanotechnology.

Even with the use of state-of-the-art BBB-penetrating biodegradable nanoparticles, *in vivo* editing still faces two challenges: the difficulty of correcting all cells, and the potential for off-target effects. These differences have led to the consideration of a complementary “edit first, transplant later” strategy, in which editing and quality control steps are performed *ex vivo* on the patient’s cells. This approach requires high-throughput correction of patient-derived induced pluripotent stem cells or neural stem/progenitor cells. A recent study has developed magnetic nanoparticle-assisted genome editing technology, which enables the precise correction of stem cells *ex vivo* and their subsequent autologous transplantation (Cho et al., 2024). Since editing is performed in culture, all clones can be tested for targeting accuracy, karyotypic integrity, and pluripotency prior to transplantation. The latest magnetic nanoparticle-assisted genome editing protocol delivers multiple plasmids with > 99% cellular uptake and about 43% allele repair in Rett syndrome neural progenitor cells, which is sufficient to restore synaptic function. After transplantation, these corrected cells not only supply functional gene products but also reconstruct damaged circuits. Achieving this outcome is challenging with gene editing alone when neuronal loss is already advanced.

**Figure 1:**
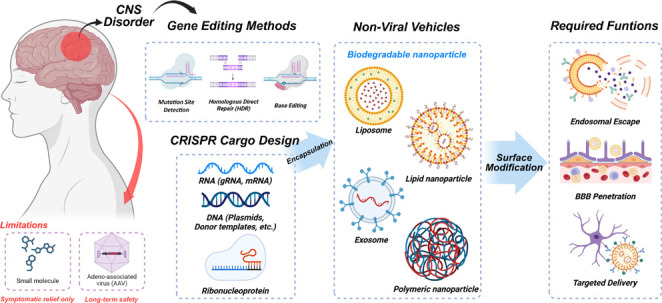
Strategy of nanoparticle-based gene therapy for CNS disorders. Created with BioRender.com. BBB: Blood–brain barrier; CNS: central nervous system; CRISPR: clustered regularly interspaced short palindromic repeats.

The treatment of CNS genetic diseases using *in vivo* nanoparticle delivery and *ex vivo* edited cell therapy creates a two-pronged approach. Nanoparticles are highly effective at quickly silencing or repairing genes in situ, while edited grafts restore the cell populations that cannot be repaired. Future clinical protocols are likely to combine these methods: stabilizing the disease with systemic nanoparticle doses, then regenerating permanently lost areas with patient-specific grafts. This strategy is expected to open new therapeutic options for conditions such as Rett syndrome, spinal muscular atrophy, and Fragile X through precision gene editing and regenerative cell therapy.

*JWC acknowledges the financial support from the National Research Foundation of Korea (NRF) grant funded by the Korea government (MSIT) (RS-2024-00344633). HYC acknowledges the financial support from the National Research Foundation of Korea (NRF) grant funded by the Korea government (MSIT) (RS-2023-00211360) and Biomaterials Specialized Graduate Program through the Korea Environmental Industry & Technology Institute (KEITI) funded by the Ministry of Environment (MOE).*
